# Miscellany

**Published:** 1890-07

**Authors:** 


### Miscellany.

### RECENT PATENTS, ETC., GRANTED ON SUBJECTS AFFECTING THE MEDICAL AND SURGICAL INTERESTS.

Reported by CHARLES J. GOOCH, Patent Attorney, Washington, D. C.

Inhaler, R. Macdonald, New York, N. Y.; Inhaler, J. O. Woods, New York, N. Y.; Inhaling apparatus, R. Macdonald, New York, N.Y.; Medicine Spoon, C. Daniclowsky, Breslau, Prussia, Germany; Sick room appliance, J. G. Thrower, Atlanta, Ga.; Syringe, H. Read, Jersey City, N. J.; Acid compound of dimothylmeta—amidophonol, R. Gnehm & J. Schmid, Basle, Switzerland; Carbonic acid compound of metha-amidophonol, R. Gnehm & J. Schmid, Basle, Switzerland; Recovering glycerine from spent soap lye, A. Domeier & O. C. Haggemann, London, England; (1) Recovering glycerine from spent soap lye, E. K. Mitting, Chicago Ill.; (2) Recovering glycerine from spent soap lye, E. K. Mitting, Chicago, Ill.; Purifying glycerine, O. C. Haggemann, London, England; Distilling glycerine recovered from spent soap lye, A. Domeier & O. C. Haggemann, London, England; Liniment, S. J. Lancaster, Petrolia, Ontario, Canada; Head-band for surgical mirrors, J. L. Sardy, New York, N. Y.

### TRADE-MARKS.

Antiseptic medical substance in powdered form (2), Farbenfabriken, vormals, Fr. Bayer & Co., Elberfeld, Germany; Hypnotic medicine,

H. K. Wampole & Co., Philadelphia, Pa.; Solid medicines usually in the form of lozenges, tablets and troches, Choate Drug and Chemical Co., Boston, Mass.; Anti-gonorrheic and adstringous medicines, Farbenfabriken, vormals, Fr. Bayer & Co., Elberfeld, Germany; Antiseptic astringents, E. T. Jester & Co., St. Louis, Mo.; Balm having both cosmetic and remedial qualities, C. J. Wilkins, Denver, Colo.; Emulsion of cod liver oil, J. A. Magee, Lawrence, Mass.; Liniment, W. W. Watkins, Kansas City, Mo.; Medical drugs, W. H. Schieffelin & Co., New York, N. Y.; Blood-purifying medicine, A. N. Callaway & C. O. McClure, San Antonie, Tex.; Medicine for kidney and heart diseases, Knoll & Co., Ludwigshafen-on-the-Rhine, Germany; Certain named medicines, A. P. & M. I. Partridge, St. Paul, Minn.; Medical tonic (2), W. Kalle, Biebrich, Germany; Remedy for nervous diseases, S. A. Richmond, Tuscola, Ill.; Remedy for pulmonary diseases, A. M. Chase, Orleans, Mass.; Medical compound for the certain named diseases, F. A. & C. B. Dicks, Natchez, Miss.; Cod liver oil, Scott & Bowne, New York, N. Y.

LABELS.

F. R. Hanrath, Cleveland, Ohio—(Medicinal Tinctures): Apis, Apocynum, Baptisia, Belladonna, Bryonia, Cactus, Carbo Veg., Chelidonium, Collinsonia, Cuprum, Drosera, Eryngium, Gelseminum, Impecacuanka, Lobelia, Macrotys, Nux Vomica, Phytolacca, Podophyllum, Pulsatilla, Rhus, Sticta, Veratrum, Viburnum; Bold's Tooth Powder, L. H. Bold & C. Jareckie, New York, N. Y.; Dr. James' Cherry Tar Syrup, J. W. James & Co., Brady's Bend, Pa.; Lessor Gardner's Cough Balsam, L. Gardner, Stanfordville, N. Y.; Stackhouse's Family Cough Syrup, J. Stackhouse, Philadelphia, Pa.; Magee's Emulsion of Pure Cod Liver Oil in combination with Extract of Malt and Hypophosphites of Lime and Soda, J. A. Magee, Lawrence, Mass.; Stackhouse's Liver and Anti-Malaria Pills, J. Stackhouse, Philadelphia; Blood Purifier and Rheumatic Cure, S. E. McCormick, Lancaster, Pa.; Day's Dyspepsia Discs (for a medicine), W. L. Day, New York, N. Y.; Glycerite of Menthol, neuralgic headache, and toothache cure, P. A. Turner & H. C. Ross, Pittsburg, Pa.; M. A. Curtis, Herb Ointment, M. A. Curtis, San Francisco, Cal.; Marshmallow Lotion (for the skin), Diamond Laboratory Co.; Marshmallow Throat and Lung Tablets, Diamond Laboratory Co., Penn., L. Naugatuck, Conn.; Neilson's Diarrhea Cure, F. L. Jacobs, Asheville, N. C.

CALF-PEPSIN.—Dr. Frank Woodbury, who introduced the glycerite of calf-pepsin, has an article in the *Medical Bulletin* for June, 1890, advocating its adoption by the United States Pharmacopeia, which at the last revision admitted hog pepsin, but acknowledged no other kind. In the case of infants, and in patients upon a milk diet, calf-pepsin is more appropriate, as it affords the physiological aid to digestion.

---

THE Maltine Manufacturing Company, of New York, has just published Part VI. of the series of brochures that this house issued at irregular though frequent periods. This number is entitled "Maltine with Pepsin and Pancreatine," and contains also the indorsements of many of the profession with reference to this combination of nutritive and digestive agents. The complete set of Maltine publications can be obtained on application to the company, 19 Warren street, New York.

---

THE HARDERFOLD FABRIC Co., of Troy, N. Y., manufactures a hygienic underwear that is not excelled by any with which we are familiar. The celebrated Jaeger underwear that has so long held sway with many must henceforth look to its laurels, for the Harderfold is certainly superior to it in many respects in our view, and we speak from a personal experience with both. It is a two-fold fabric throughout; that is, it is double, thereby giving an inter-air space and a smooth surface next to the skin. It is sold by leading merchants in the principal cities, and we advise a trial of it by those about to purchase.

---

HOW WOMEN SHOULD SIT.—Women who sit with their legs crossed, to sew or to read or to hold the baby, are not aware that they are inviting serious physical ailments; but it is true nevertheless. When a man crosses his legs he places the ankle of one limb across the knee of the other, and rests it lightly there. A woman, more modest and restricted in her movements, rests the entire weight of one limb on the upper part of the other, and this pressure upon the sensitive nerves and cords, if indulged in for continued lengths of time, as is often done by ladies who sew or embroider, will produce disease. Sciatica, neuralgia, and other serious troubles frequently result from this simple cause. The muscles and nerves in the upper portion of a woman's legs are extremely sensitive, and much of her whole physical structure can become deranged if they are overtaxed in the manner referred to.—*Ladies' Home Journal.*

TARRANT'S SELTZER APERIENT is an effervescent alkaline salt of great value in lithemia. It is a valuable remedy in habitual constipation, and is an efficient vehicle for the administration of many iron preparations. Moreover, it is agreeable and wholesome as a daily medicine where alkalies are demanded in many urinary affections. Its general use by the profession has not been disappointing, and it has already become standard in the directions named.

---

PARKE, DAVIS & Co. are out this month with some seasonable suggestions as to eligible remedies for prevalent diseases of hot weather. They have a very convenient list of intestinal sedatives, antiseptics, antispasmodics and anodynes for diarrheal and dysenteric affections, some new expectorants of note for coughs and colds, and a normal liquid ipecac always reliable as an emetic in cases of gastric disturbances due to accumulated fermented food, so frequent a cause of infantile diarrhea. We may also state that this house is largely increasing its facilities for the manufacture of Pharmaceuticals. Buildings now in process of erection will double their capacity for production this year, and a new laboratory, very complete in its appointments, is now being built for them in Canada.

---

J. B. LIPPINCOTT COMPANY announce in press an important work on Regional Anatomy in its Relation to Medicine and Surgery, by George McClellan, M. D., Lecturer on Descriptive and Regional Anatomy at the Pennsylvania School of Anatomy, Professor of Anatomy at the Pennsylvania Academy of Fine Arts, Member of the Association of American Anatomists, Academy of Natural Sciences, Academy of Surgery, College of Physicians, etc., of Philadelphia, with about 100 full-page fac-simile illustrations reproduced from photographs taken by the author of his own dissections, expressly designed and prepared for this work, and colored by him after nature; to be complete in two large quarto volumes of 250 pages each. The object of this work is to convey a practical knowledge of regional anatomy of the entire body, the text to embrace, besides a clear description of the parts in systematic order, the most recent and reliable information regarding anatomy in its medical and surgical relations. The illustrations are intended to verify the text, and to bring before the reader the parts under consideration in as realistic a manner as possible. Volume I. will be ready for publication about December 1st, and the second volume is expected to appear shortly thereafter. The work will be sold by subscription only, and salesmen will begin an active canvass the coming October.

